# A sono-responsive antibacterial nanosystem co-loaded with metformin and bone morphogenetic protein-2 for mitigation of inflammation and bone loss in experimental peri-implantitis

**DOI:** 10.3389/fbioe.2024.1410230

**Published:** 2024-05-24

**Authors:** Bo Hu, Wang Qiao, Yang Cao, Xiaoming Fu, Jinlin Song

**Affiliations:** ^1^ College of Stomatology, Chongqing Medical University, Chongqing, China; ^2^ Chongqing Key Laboratory of Oral Diseases, Chongqing, China; ^3^ Chongqing Municipal Key Laboratory of Oral Biomedical Engineering of Higher Education, Chongqing, China; ^4^ Department of Stomatology, Shapingba Hospital Affiliated to Chongqing University, Chongqing, China; ^5^ Chongqing Key Laboratory of Ultrasound Molecular Imaging, Institute of Ultrasound Imaging, The Second Affiliated Hospital of Chongqing Medical University, Chongqing, China

**Keywords:** sono-responsive nanosystems, ultrasound stimulation, antibacterial, peri-implantitis, anti-inflammatory, bone repair

## Abstract

**Background:**

Dental implants have become an increasingly popular option for replacing missing teeth, and the prevalence of peri-implantitis has also increased, which is expected to become a public health problem worldwide and cause high economic and health burdens. This scenario highlights the need for new therapeutic options to treat peri-implantitis.

**Methods:**

In this study, we proposed a novel sono-responsive antibacterial nanosystem co-loaded with metformin (Met) and bone morphogenetic protein-2 (BMP-2) to promote efficacy in treating peri-implantitis. We introduced the zeolitic imidazolate framework-8 (ZIF-8) as a carrier for hematoporphyrin monomethyl ether (HMME) to enhance the antibacterial effect of sonodynamic antibacterial therapy and tested its reactive oxygen species (ROS) production efficiency and bactericidal effect *in vitro*. Afterward, HMME-loaded ZIF-8, BMP-2-loaded polylactic acid-glycolic acid (PLGA), and Met were incorporated into gelatin methacryloyl (GelMA) hydrogels to form HMME@ZIF-8/Met/BMP-2@PLGA/GelMA composite hydrogels, and the biocompatibility of which was determined *in vitro* and *in vivo*. A bacterial-induced peri-implantitis model in the maxilla of rats was established to detect the effects of the composite hydrogels with adjunctive use of ultrasound on regulating inflammation and promoting bone tissue repair *in vivo*.

**Results:**

The results indicated that HMME@ZIF-8 with ultrasound stimulation demonstrated more better ROS production efficiency and antimicrobial efficacy. The composite hydrogels had good biocompatibility. Ultrasound-assisted application of the composite hydrogels reduced the release of the inflammatory factors IL-6 and TNF-α and reduced bone loss around the implant in rats with bacterial-induced peri-implantitis.

**Conclusion:**

Our observations suggest that HMME@ZIF-8 may be a new good sonosensitizer material for sonodynamic antibacterial therapy. The use of HMME@ZIF-8/Met/BMP-2@PLGA/GelMA composite hydrogels in combination with ultrasound can provide a novel option for treating peri-implantitis in the future.

## 1 Introduction

Loss of teeth is a common oral problem in humans that can compromise pronunciation, chewing, aesthetics and so on, resulting in poor quality of life. Dental implantation is one of the restorative methods used to replace missing teeth (Li Q. et al., 2019; Sun et al., 2020). In recent years, with the extensive development of implant restoration technology, the number of dental implants in the population has increased, and the prevalence of peri-implant inflammation has also increased, which is expected to become a public health problem worldwide and cause a high economic and health burden (Canullo et al., 2023). Peri-implantitis is a plaque-related pathological disease that occurs around an implant and is characterized by peri-implant mucosal inflammation and loss of supporting bone (Berglundh et al., 2018). There are many risk factors for peri-implantitis, including smoking, moderate to severe periodontitis, dental plaques, poor implant positions, defective repair types, and other factors (Romandini et al., 2021). Moreover, the imbalance of oral flora and the attachment of dental plaques are the main pathogenic factors leading to peri-implant inflammation and even implant loss (Shi et al., 2023). Peri-implantitis is considered the most challenging biological complication of implantation (Cheng et al., 2023). Without systematic treatment, the disease may continue to progress and eventually cause the implant to loosen or fall off (Berglundh et al., 2019). Currently, the clinical treatment of peri-implantitis can be divided into surgical treatment (resective and regenerative surgery) and nonsurgical treatment (mechanical, chemical, antibiotic, laser methods and oral hygiene education) (Boccia et al., 2023). Plaque removal and control infection are the main aim of nonsurgical treatment, but the outcome of nonsurgical treatment of peri-implantitis is unpredictable. Surgical treatment involves removal of granulation tissue, reduction or removal of pathological peri-implant pockets, or performance of guided bone regeneration (GBR) surgery to address the problem of bone resorption. However, peri-implantitis surgery is complicated, the associated trauma is high, and the curative effect is not exact (La Monaca et al., 2018; Carcuac et al., 2020). After surgery, gingival recession, implant neck exposure, and decreased self-cleaning can occur, thus resulting in plaque biofilm attachment and formation and secondary infection (Schwarz et al., 2021). Therefore, it is necessary not only to remove plaque from the implant structure but also to administer continuous antibacterial treatment in the later stage to prevent plaque microorganisms from recolonizing the structure (de Tapia et al., 2019). Anti-infective treatment is an important aspect of peri-implantitis treatment.

At present, the antibacterial treatment of peri-implantitis mainly includes mechanical treatment, drug treatment, and photoacoustic dynamic treatment (Zhao T. et al., 2022). Antimicrobial photodynamic therapy (PDT) has been applied in the clinical treatment of peri-implantitis. The photochemical reaction between a laser and a photosensitizer produces reactive oxygen species (ROS), which can achieve bactericidal effects (Liu W. et al., 2023). However, the penetration of lasers and drugs is relatively limited (Wang Y. et al., 2022). During peri-implant infection, microorganisms typically invade deep tissues and form biofilms, which are difficult to completely remove via PDT. In contrast, ultrasound is a mechanical sound wave that penetrates deeper into soft tissues than light and can exert anti-infection effects through the targeted activation of sonosensitizers (Son et al., 2020; Zhang et al., 2021). In addition, ultrasound can enhance drug efficacy by facilitating drug delivery in human tissues and biofilms through cavitation effects (Roovers et al., 2019). Sonodynamic therapy (SDT) is considered a new noninvasive anti-infective treatment method, and the sonosensitizer is activated by using acoustic energy to generate ROS, which kill microorganisms (Rwei et al., 2017; Xu Q. et al., 2021). The production of ROS in SDT is an essential step in the killing of bacteria; thus, it is necessary to promote the formation of ROS. Zeolitic imidazolate framework-8 (ZIF-8) is a representative metal-organic framework comprising zinc ions and 2-methylimidazolium salts (Zhao S. et al., 2022); it is a porous nanomaterial possessing characteristics of high porosity, large specific surface area and adjustable functional surface (Chen et al., 2022). The advantages of ZIF-8, such as its high porosity and high specific surface area, enable it to improve its drug loading efficiency. In recent years, it has been widely used as a drug carrier for treating tumors (Siboro et al., 2022), atherosclerosis (Obaid et al., 2022), inflammation, antibacterial agents and other conditions (Zhang et al., 2022). Moreover, ZIF-8 also has the advantages of excellent gas adsorption and storage and can enhance the ultrasonic cavitation effect. The enhanced ultrasonic cavitation effect can promote the formation of ROS (Li Y. et al., 2022). Therefore, ZIF-8 may be a good candidate material for use as an ultrasonic responsive carrier. However, the ROS production efficiency and bactericidal effect of the introduction of ZIF-8 into sonodynamic antibacterial therapy are unclear and require further investigation.

In addition, the treatment principles for peri-implantitis include not only the continuous removal of plaques around the implant to remove pathogenic factors but also the regulation of inflammation, prevention of bone loss, and promotion of bone regeneration (Carcuac et al., 2017). The persistent chronic inflammatory state of the tissue around the implant will lead to destruction of the alveolar bone around the implant (Mijiritsky et al., 2019). Therefore, regulating the inflammatory state after antibacterial treatment and promoting bone tissue repair will contribute to the long-term stability of the implant. However, current sonodynamic antibacterial therapies mainly focus on antibacterial efficacy, whereas combined therapies involving the regulation of inflammation and the promotion of bone regeneration are rarely considered. Therefore, promoting inflammation regulation and bone repair effects while enhancing the anti-infection effect of SDT is considerably important for peri-implantitis treatment and merits further research. Metformin (Met), which is the most widely prescribed drug for lowering blood glucose in individuals with type 2 diabetes, has recently been considered a promising anti-inflammatory agent (Wen et al., 2019). Increasing evidence shows that Met has a significant protective effect on a variety of inflammatory diseases, including myocarditis (Liu et al., 2017), colitis (Xiong et al., 2021), hepatitis (Xu L. et al., 2021), arthritis (Li et al., 2020), and periodontitis (Zhao X. et al., 2022). Several clinical trials have shown that the use of Met, which is an adjuvant therapy after root curettage, can reduce the modified gingival sulcus bleeding index and probing depth, as well as increase bone defect repair and clinical attachment (Pradeep et al., 2013; Rao et al., 2013; Pradeep et al., 2017; Pankaj et al., 2018). Bone morphogenetic protein (BMP) is a naturally occurring protein that plays an important regulatory role in the process of bone regeneration and repair, and BMP-2 is an important osteogenic regulator that can strongly induce osteogenesis (Guo et al., 2023). The auxiliary application of BMP-2 has been used clinically to improve bone regeneration. The United States Food and Drug Administration has approved the use of recombinant human BMP-2 for maxillary sinus elevation and bone graft surgery related to extraction sockets (Kelly et al., 2016). However, to date, there has been no research on the treatment of peri-implantitis by using sonodynamic antibacterial therapy combined with Met and BMP-2.

**FIGURE 1:**
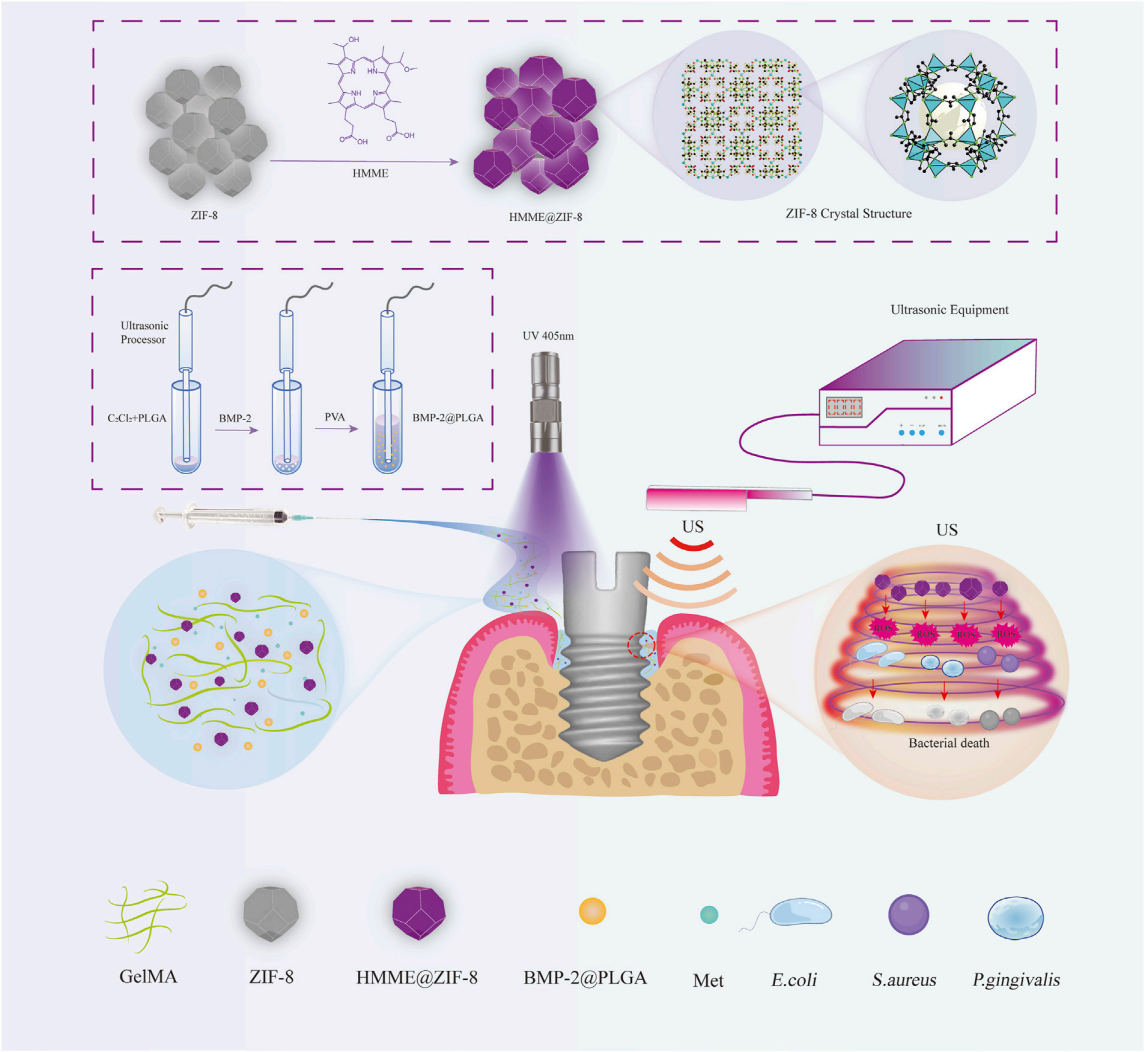
Schematic illustration of the HMME@ZIF-8/Met/BMP-2@PLGA/GelMA composite hydrogels with ultrasound assistance for the treatment of peri-implantitis.

Therefore, we propose a novel therapy for peri-implantitis involving antibacterial, anti-inflammatory, and bone repair promotion based on SDT by developing a new sono-responsive nanosystem. We first introduced ZIF-8 as a carrier for the sonosensitizer hematoporphyrin monomethyl ether (HMME) to enhance the antibacterial effect of sonodynamic antibacterial therapy and tested the ROS production efficiency and bactericidal effect of HMME@ZIF-8 *in vitro*. Afterward, HMME-loaded ZIF-8, BMP-2-loaded polylactic acid-glycolic acid (PLGA), and Met were incorporated into gelatin methacryloyl (GelMA) hydrogels to form HMME@ZIF-8/Met/BMP-2@PLGA/GelMA composite hydrogels, and the biocompatibility of which was determined *in vitro* and *in vivo*. Additionally, a bacterial-induced peri-implantitis model in the maxilla of rats was established to detect the effect of the composite hydrogels with the adjunctive use of ultrasound on regulating inflammation and promoting bone tissue repair *in vivo*, thus providing a theoretical basis for the application of sono-responsive nanosystems with ultrasound assistance in peri-implantitis treatment in the future (Figure 1).

## 2 Materials and methods

### 2.1 Materials

HMME, methanol, isopropanol, and dichloromethane (CH_2_Cl_2_) were purchased from Shanghai Macklin Biochemical Technology Co., Ltd. (Shanghai, China). ZIF-8 and PLGA (lactide:glycolide = 50:50, PLGA = 45,000 Da MW) were purchased from Ruixi Biotechnology Co., Ltd. (Xi’an, China). Normal saline, phosphate buffer saline (PBS), and Met were obtained from Beijing Solarbio Sciences Co., Ltd. (Beijing, China). Polyvinyl alcohol (PVA) and 1,3-diphenylisobenzofuran (DPBF) were obtained from Sigma–Aldrich Co. (Missouri, United States). GelMA hydrogels and lithium phenyl-2,4,6-trimethylbenzoylphosphinate (LAP) were purchased from Yongqinquan Intelligent Equipment Co., Ltd. (Suzhou, Jiangsu, China). *Escherichia coli* (*E. coli*), *Staphylococcus aureus* (*S. aureus*) and *Porphyromonas gingivalis* (*P. gingivalis*) were purchased from ATCC Co. (Minnesota, USA). Mouse calvaria-derived osteoblast-like cells (MC3T3-E1) were purchased from Hunan Fenghui Biotechnology Co., Ltd. (Changsha, Hunan, China). Pure titanium microimplants were purchased from Dongguan Genrui Precision Technology Co., Ltd. (Dongguan, Guangzhou, China). The Sprague–Dawley rats that were used in this study were purchased from the Chongqing Medical University Animal Care and Use Committee. All of the reagents in this study were of analytical grade and were used as received.

### 2.2 Preparation of HMME@ZIF-8/Met/BMP-2@PLGA/GelMA composite hydrogels

#### 2.2.1 Synthesis of HMME@ZIF-8

ZIF-8 (50 mg dispersed in 15 mL of methanol), methanol (10 mL), and HMME (5 mg dissolved in 5 mL of methanol) were mixed and stirred with a magnetic stirrer (100 rpm for 12 h) at room temperature. After mixing, the mixture was washed with ultrapure water, and the products were separated via centrifugation and washed with ultrapure water three times (10,000 rpm, 15 min). The final product was obtained by drying. After sample synthesis, part of the powder was extracted on the sample platform; after vacuum drying and gold spraying, the microscopic morphology was observed using scanning electron microscopy (SEM, JSM-7800F, JEOL, Japan). The particle size of HMME@ZIF-8 was measured via dynamic light scattering (DLS, Malvern Instruments, UK). The characteristic absorption peak was measured by using a UV–Vis spectrophotometer (UV2500, Techcomp, Shanghai, China).

#### 2.2.2 Synthesis of BMP-2@PLGA

BMP-2@PLGA nanoparticles were prepared by using the double emulsion method (Zhu et al., 2020). Initially, 50 mg of PLGA was dissolved in 2 mL of dichloromethane (oil phase: O), and 10 μg of BMP-2 was added to sterile ultrapure water (water phase: W). The BMP-2 solution was subsequently added to the dichloromethane solution (water/oil: W/O), followed by acoustic vibration using an acoustic vibrato for 3 min under ice bath conditions. Subsequently, 8 mL of 4% PVA solution (water/oil/water: W/O/W) was added, and a second round of acoustic vibration was performed. Afterward, ultrapure water containing 2% isopropanol was added, and the mixture was stirred for 2 h with a magnetic stirrer. After complete volatilization of the dichloromethane, the BMP-2@PLGA nanoparticles were prepared via centrifugation, washed three times with ultrapure water (at 10,000 r/min for 5 min each time) and subsequently stored at 4 °C. Following synthesis, the samples were dispersed in ultrapure water, and 5 μL was dropped onto the cover glass. After 24 h at room temperature, the samples were vacuum dried and sprayed with gold, and the microstructure was observed via SEM. The particle size of the BMP-2@PLGA NPs was measured by using DLS.

#### 2.2.3 Fabrication of HMME@ZIF-8/Met/BMP-2@PLGA/GelMA composite hydrogels

HMME@ZIF-8, BMP-2@PLGA, and Met were added to the GelMA hydrogel precursor solution at a mass ratio of 2:5:5 and stirred for 10 min. The resulting nanocomposite solution exhibited the ability to undergo a sol‒gel transition under UV light. Subsequently, composite hydrogels loaded with HMME@ZIF-8, Met, and BMP-2@PLGA were formed. The shear-thinning properties of the composite hydrogels were tested *in vitro* by using a rheometer (MCR 302, Anton Paar, Austria), and a vial inversion experiment was conducted.

### 2.3 *In vitro* ROS production testing

The ROS generation of HMME@ZIF-8 via ultrasound activation was assessed using DPBF, as previously described (Yao et al., 2021). Briefly, a mixture of DPBF and HMME@ZIF-8 nanoparticles was irradiated with low-intensity continuous ultrasound (1.5 W/cm^2^, 1 MHz, National Engineering Research Center of Ultrasound Medicine, Chongqing Medical University, China) for 120 s in the dark. Subsequently, the absorbance and consumption of DPBF were measured by using a UV–Vis spectrophotometer. The ROS production of HMME and ZIF-8 after ultrasound irradiation was measured in the same manner.

### 2.4 *In vitro a*ntibacterial effect assay

*E. coli*, *S. aureus* and *P. gingivalis* were selected for evaluating the bactericidal effect of HMME@ZIF-8 under ultrasound irradiation *in vitro*. The colony forming unit (CFU) counting method was used to quantitatively evaluate the antibacterial effect on the different strains (Khosalim et al., 2022). The diluted bacterial broths of different bacteria were added to equal volumes of solutions containing HMME@ZIF-8, ZIF-8 or HMME. The final concentration of these bacterial solutions in each group was 1 × 10^−6^ CFU mL^-1^. The final concentrations of HMME@ZIF-8, ZIF-8 and HMME in these bacterial solutions were 200 μg/mL, 200 μg/mL, 50 μg/mL, respectively. Afterward, then ultrasonic irradiation (30 mW/cm^2^, 1 MHz) was performed for 30 min, and the control group was left for static treatment. The final 100 μL of bacterial mixture was aspirated from each sample, diluted in a gradient and transferred to LB agar plates and Columbia blood agar plates for colony counting to calculate the bactericidal rate of each group.

### 2.5 *In vitro* drug release studies

Cumulative drug release analysis of Met and BMP-2 from the hydrogels was conducted. A mixture of 0.5 mg of Met and 1 mL of GelMA hydrogel precursor solution was prepared, followed by curing the GelMA hydrogel precursor solution under ultraviolet light irradiation for 60 s. Subsequently, sterile PBS was added, and the mixture was incubated at 37 °C. After liquid removal at predetermined time points (8, 16, 24, 48, 72, and 96 h), an equal volume of fresh PBS was added. The concentration of Met in the removed solution was determined by using UV–Vis spectroscopy at a wavelength of 233 nm, with the hydrogel without Met serving as a blank control. The release curve was calculated based on the standard curve established for Met. When considering the cost of BMP-2, BSA was used as a model drug to simulate protein drug release according to a previous study (Li D. et al., 2022). BSA@PLGA was fabricated in the same manner as BMP-2@PLGA. The release conditions and liquid collection process for BSA were identical to those described above. The protein concentration in the collected liquid samples was measured by using a BCA protein concentration assay kit (Beyotime Biotechnology, Shanghai, China) to calculate the cumulative release curve.

### 2.6 *In vitro* biocompatibility investigation

To quantitatively assess the biocompatibility of the composite hydrogels *in vitro*, cytotoxicity studies were performed on MC3T3-E1 cells. The six groups of hydrogels (0, 25, 50, 150, 100, and 200 mg) were incubated in complete medium (DMEM supplemented with 10% fetal bovine serum, 100 mg/mL streptomycin, and 100 U/mL penicillin) for 24 h at 37 °C in an incubator with 5% CO_2_ to obtain the extract. Confluent MC3T3-E1 cells were digested, seeded in 96-well plates at 2×10^3^ cells/well and incubated for 24 h. After 24 h, the mixture was replaced with the composite hydrogel extract or fresh complete medium, after which the cells were incubated for 1, 2, 3, or 4 days. Cell viability was determined by utilizing a Cell Counting Kit-8 (CCK-8, Solarbio, Beijing, China) at each timepoint. The absorbance values were read at a wavelength of 450 nm by using a multifunctional microplate reader (Perkin Elmer, United States).

### 2.7 *In vivo* biocompatibility, inflammatory regulation and bone repair assays in a bacterial-induced peri-implantitis rat model

This study was approved by the Ethics Committee of the Stomatology School of Chongqing Medical University (No. 2023-013), and all of the experimental procedures were conducted in accordance with the principles outlined in the Declaration of Helsinki. Male Sprague‒Dawley rats (8-weeks-old, weighing 250–300 g) were randomly distributed to the control, peri-implantitis, peri-implantitis + GelMA, and peri-implantitis + HMME@ZIF-8/Met/BMP-2@PLGA/GelMA + ultrasound (US) groups. First, a bacterial-induced peri-implantitis model in the maxilla of rats was established. Briefly, after anesthesia was administered, the maxillary first molar was loosened by using gentle force and completely extracted while the root was intact in all of the rats. A pilot hole with a width of 1.6 mm and a depth of 2.5 mm was drilled under continuous irrigation with sterile PBS solution. Pure titanium microimplants (diameter of 2 mm, length of 4.5 mm) were immediately placed at the drilling site, which had good primary stability. Apart from the control group, the bacterial solution of *P. gingivalis* (OD_600_ = 1) was injected around the microimplant in all of the other groups once every 2 days for 2 weeks. Two weeks later, bleeding on probing, ulceration, and necrosis were clinically observed in the tissue surrounding the microimplants.

Subsequently, the HMME@ZIF-8/Met/BMP-2@PLGA/GelMA composite hydrogels were injected around the implant by using a microsyringe and irradiated with a light-curing lamp for 60 s, after which the hydrogels were changed from sol to gel, and the site of these rats was exposed to ultrasound irradiation (2.4 W/cm^2^, 1 MHz) for 5 min. One week later, IL-6 and TNF-α expression in the gingival tissues around the implants was detected via enzyme-linked immunosorbent assay (ELISA) with an IL-6 ELISA kit (Boster Biological Technology, Wuhan, China) and a TNF-α ELISA kit (Thermo Fisher Scientific, Waltham, United States). Four weeks later, blood samples were collected from the eyeballs for routine blood tests and liver and kidney function tests; additionally, the main organs of the heart, liver, spleen, lung and kidney were harvested for histological sectioning and H&E staining. All of the rats were euthanized by using CO_2_ asphyxiation when the animals were sacrificed. The maxillae were harvested, fixed with 4% paraformaldehyde and scanned via micro-CT. The maxillary samples were subsequently decalcified with 10% EDTA for 1 month. After decalcification, the samples were sectioned along the sagittal plane for H&E staining and Masson staining.

### 2.8 Statistical analysis

OriginPro2021 was used for the statistical analysis and plotting of the experimental data, and all of the data are presented as the means ± standard deviations. The significance of differences between samples was determined by using one-way ANOVA. Statistical significance was defined as *p < 0.05, **p < 0.01, and ***p < 0.001.

## 3 Results and discussion

### 3.1 Characterization of the HMME@ZIF-8/Met/BMP-2@PLGA/GelMA composite hydrogels

**FIGURE 2:**
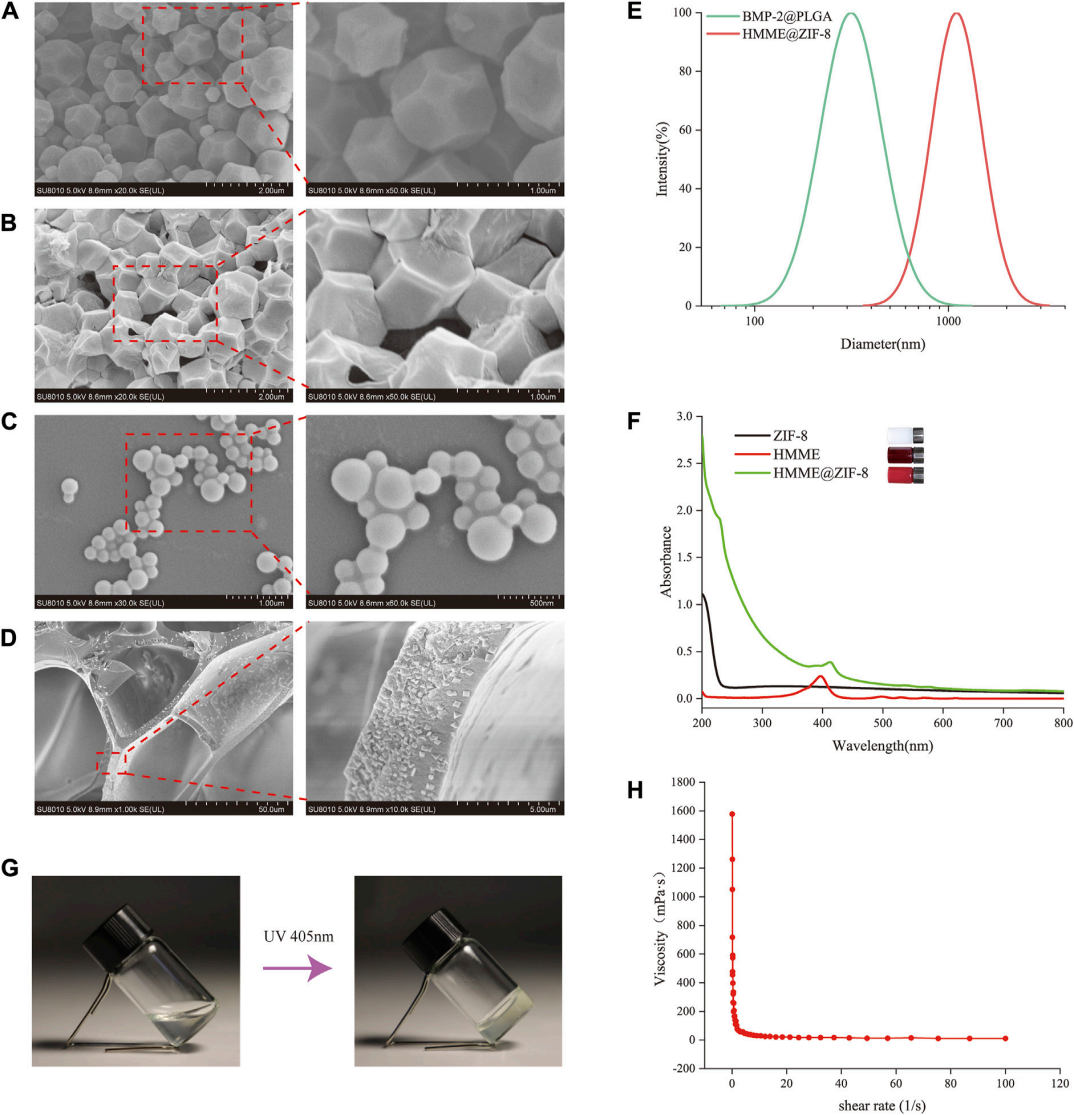
Characterization of the HMME@ZIF-8/Met/BMP-2@PLGA/GelMA composite hydrogels. **(A, B)** SEM images of ZIF-8 and HMME@ZIF-8. Scale bars = 2 μm, 1 μm. **(C)** SEM images of BMP-2@PLGA. Scale bars = 1 μm, 500 nm. **(D)** SEM images of HMME@ZIF-8/Met/BMP-2@PLGA/GelMA. Scale bars = 50 μm, 5 μm. **(E)** Size distributions of BMP-2@PLGA and HMME@ZIF-8. **(F)** UV–visible absorption spectra of ZIF-8, HMME and HMME@ZIF-8. **(G)** Sol-gel conversion of HMME@ZIF-8/Met/BMP-2@PLGA/GelMA. **(H)** Viscosity-shear rate curve of HMME@ZIF-8/Met/BMP-2@PLGA/GelMA.

HMME, which is a new generation of sonosensitizers, can produce ROS under the activation of ultrasound to achieve sonodynamic antibacterial effects (Zhang et al., 2020). The metal-organic framework nanomaterial ZIF-8 may be a good candidate for use as an ultrasonic response carrier because of its numerous advantages. To enhance the antibacterial effect of SDT, ZIF-8 was introduced as a carrier for HMME in this study. ZIF-8 has a porous structure and a large specific surface area, which endows it with a strong drug-loading ability; it also has the advantages of excellent gas adsorption and storage and can enhance the ultrasonic cavitation effect. ZIF-8 has high thermal stability and corrosion resistance and is very stable in neutral aqueous solution. In addition, the good antibacterial property of Zn^2+^ in ZIF-8 further improves the sustained antibacterial ability of the material (Liang et al., 2021). ZIF-8 was white, and the HMME-loaded ZIF-8 turned pale purple. SEM images showed that ZIF-8 had a polyhedral structure with clear edges and corners before drug loading (Figure 2A), while the morphology of the ZIF-8 decreased and became wrinkled after drug loading (Figure 2B). The particle size and distribution of HMME@ZIF-8 were further determined via DLS, and the average particle size of HMME@ZIF-8 was approximately 1,300 nm (Figure 2E). The characteristic absorption peak of HMME in the UV‒visible spectrum of HMME@ZIF-8 appeared near 412 nm (Figure 2F), which further confirmed that the HMME was successfully loaded into ZIF-8. HMME@ZIF-8 nanoparticles were successfully prepared in this study and were used for subsequent experiments.

PLGA is a polymeric synthetic material with good biodegradability, good biocompatibility, and sustained release; additionally, it is widely used in drug delivery and tissue engineering (Su et al., 2021). In the present study, BMP-2@PLGA nanospheres were prepared by using the double emulsion method. Our results showed that the BMP-2@PLGA nanospheres were spherical in shape and similar in size (Figure 2C). The average particle size was approximately 200 nm (Figure 2E). All of the samples exhibited a relatively uniform particle size distribution.

GelMA hydrogels are novel materials that can be crosslinked and cured via gelation by ultraviolet or visible light under the action of a photoinitiator; moreover, they have a three-dimensional structure that is suitable for cell growth and differentiation, excellent biocompatibility, and adjustable mechanical properties and are widely used in the field of tissue engineering and regenerative medicine (Li et al., 2023). In this study, HMME@ZIF-8 and BMP-2@PLGA nanoparticles and Met were successfully incorporated into GelMA. Figure 2D shows a SEM image of the HMME@ZIF-8/Met/BMP-2@PLGA/GelMA composite hydrogels after freeze-vacuum drying, which demonstrated a uniform void distribution and a loose porous structure. A vial inversion experiment (Figure 2G) showed that the composite hydrogels had good photosensitivity. Before illumination, the hydrogels were liquid and mobile. After illumination at a wavelength of 405 nm for 60 s, the hydrogels were in the gel state and lost mobility. The rheological behavior of the hydrogels was further investigated by measuring their viscosity as a function of the shear rate. Figure 2H shows the curve of the viscosity of the composite hydrogels as a function of the shear rate, thus indicating that the hydrogel system has shear-thinning properties and can be delivered through a syringe before light curing, with good injectability. Thus, sol-to-gel conversion of the composite hydrogels can be quickly achieved, which can better cope with complex local structures and irregular bone defects around the implant. The GelMA-based composite hydrogels that were prepared in this study can be used for further *in vivo* experiments.

### 3.2 *In vitro* ROS production measurements

**FIGURE 3:**
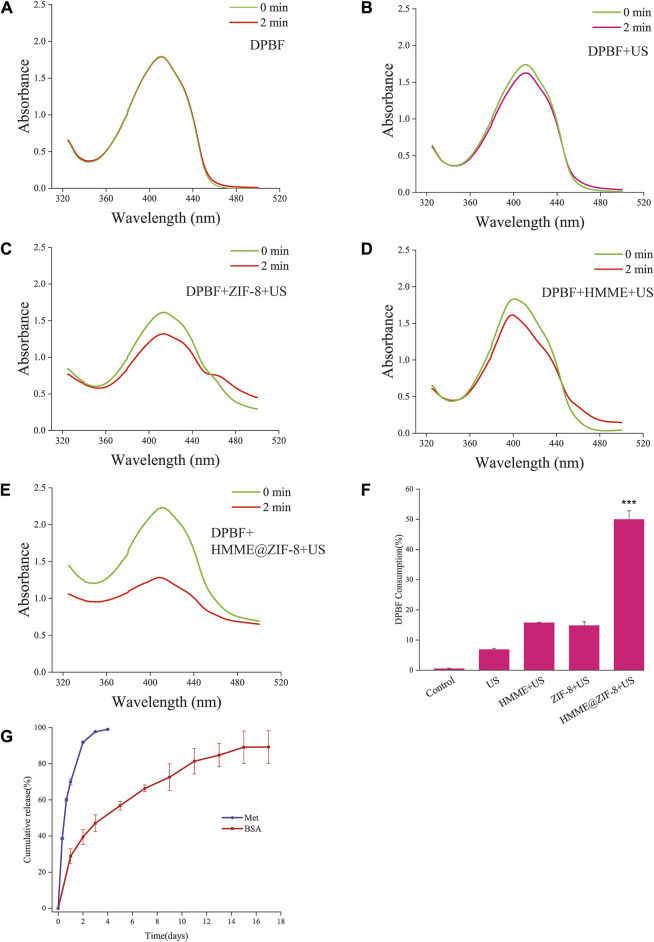
**(A–E)** Absorption spectra of DPBF with HMME, ZIF-8, and HMME@ZIF-8 after ultrasound (US) irradiation. **(F)** Quantification of DPBF consumption. **(G)** Cumulative release profiles of Met and BSA. Data are presented as the mean ± SD (n = 3). ***p < 0.001.

The production of ROS in sonodynamic antibacterial therapy is an essential step in the killing of bacteria. Therefore, in this study, the ROS production efficiency of the HMME@ZIF-8 composite was examined *in vitro*. DPBF was used as an ROS sensor, and the amount of ROS was indirectly calculated by detecting the change in the absorption intensity of DPBF at 410 nm via an ultraviolet spectrophotometer (Yao et al., 2021). Compared with that of the other groups, the absorbance of DPBF at 410 nm was significantly lower, and the consumption of DPBF was greater in the HMME@ZIF-8 with ultrasound irradiation group (Figures 3E, F). The results indicated that under ultrasound stimulation, HMME@ZIF-8 exhibited better ROS production efficiency, which may be related to the advantages of ZIF-8, such as its high porosity, high specific surface area, and excellent gas adsorption performance, which enhances the ultrasonic cavitation effect. The enhanced ultrasonic cavitation effect can promote the formation of ROS (Li Y. et al., 2022). Therefore, HMME@ZIF-8 may enhance antimicrobial efficacy (to a certain extent) by promoting ROS production in SDT.

### 3.3 Antibacterial effect assay

Bacterial infection is the major pathogenic factor leading to peri-implant inflammation and even implant loss; therefore, it is very important to reduce the number of bacteria in the peri-implant microenvironment during the treatment of peri-implantitis (Shi et al., 2023). With traditional mechanical treatment, it is difficult to completely remove pathogenic bacteria from the special thread structure at the neck of the implant. SDT can produce ROS through the activation of sonosensitizers, which have multitarget effects on bacterial cell structures and various metabolic pathways (Ren et al., 2020). The inability of bacteria to sense oxidative stress and the loss of transgenerational adaptability decrease the susceptibility of sonodynamic antibacterial therapy to bacterial resistance even after repeated treatment, and this approach has been successfully used against multidrug-resistant bacteria (Pang et al., 2019). However, previous studies have shown that HMME, which is an amphiphilic photosensitizer, is prone to aggregation due to the presence of hydrophobic groups and has poor solubility, which reduces its photoacoustic sensitivity (Wang P. et al., 2022). Moreover, these agents have the disadvantage of poor bactericidal efficacy against some Gram-negative bacteria (Hu et al., 2022; Gnanasekar et al., 2023), which may be related to the special bacterial wall structure of Gram-negative bacteria. These bacteria are more resistant than Gram-positive bacteria (Richter et al., 2017; Richter and Hergenrother, 2019). Given its numerous advantages, ZIF-8 was applied as a carrier material for HMME to improve the bactericidal efficacy of SDT. Our results showed that under ultrasound stimulation, HMME@ZIF-8 exhibited improved ROS production efficiency, and we further analyzed its bactericidal efficacy.

**FIGURE 4:**
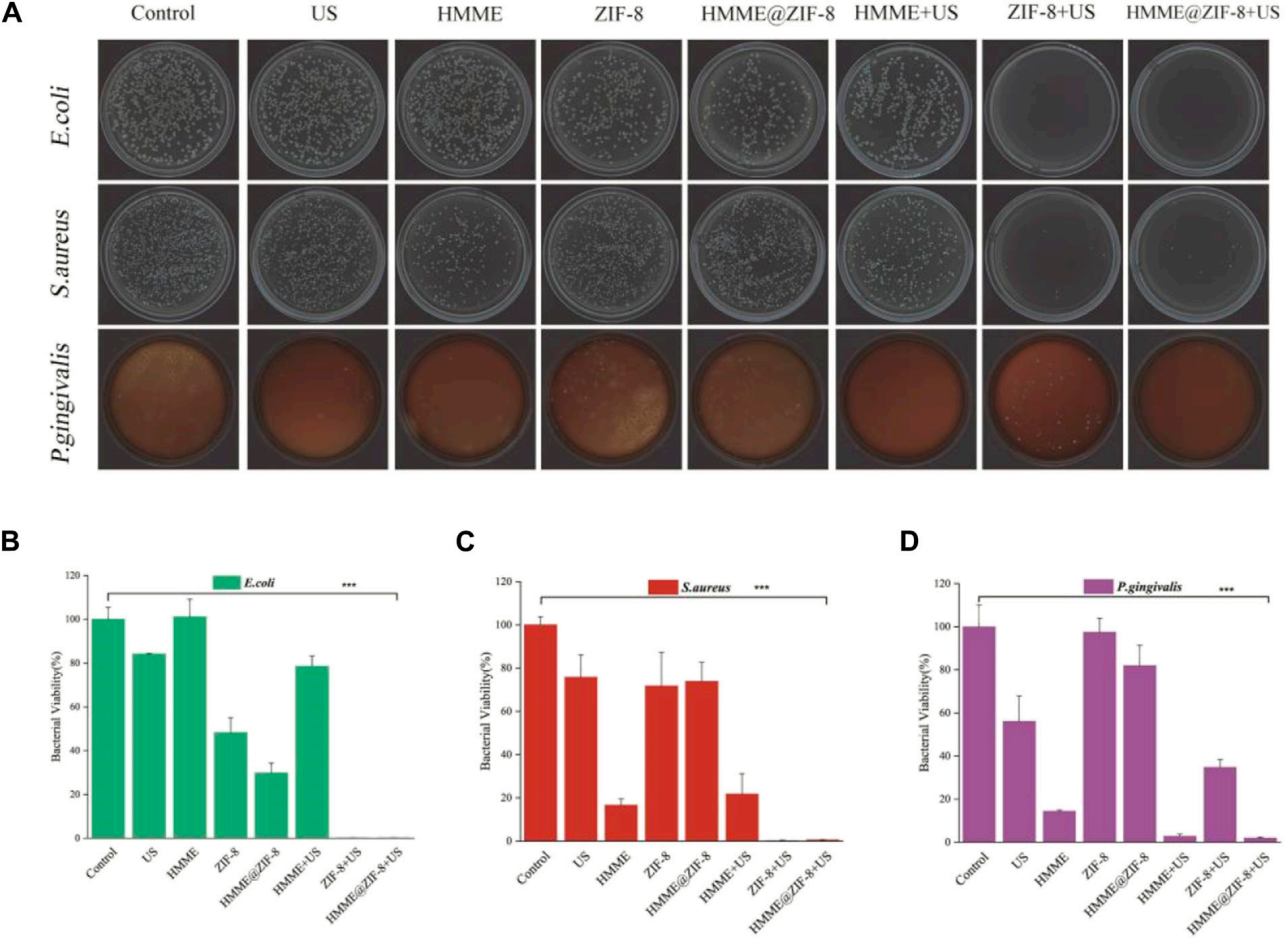
Bactericidal performance of HMME@ZIF-8 after irradiation with US. **(A)** Plate photographs of *E. coli*, *S. aureus*, and *P. gingivalis*. **(B)** Bacterial viability of *E. coli*. **(C)** Bacterial viability of *S. aureus*. **(D)** Bacterial viability of *P. gingivalis*. Data are presented as the mean ± SD (n = 3). ***p < 0.001.

In this study, the bactericidal effect of the strains was evaluated by using the CFU counting method. Three different types of bacteria, including Gram-positive bacteria (*S. aureus*), Gram-negative bacteria (*E. coli*) and anaerobic bacteria (*P. gingivalis*), were used to evaluate the antibacterial effect of HMME@ZIF-8. Our results showed that, compared with those in the control groups, the antimicrobial efficacy of the HMME@ZIF-8 group under ultrasound stimulation improved, and the HMME@ZIF-8 group showed significant killing of all three different types of bacteria (Figure 4A–D). The three types of bacteria were obviously killed in the group of pure ZIF-8 after ultrasonic treatment. After the introduction of ZIF-8 as an HMME carrier, the killing effect on *E. coli* and *S. aureus* was significantly greater than that of SDT using HMME alone (Figure 4B, C), and the killing effect on *P. gingivalis* was significantly greater than that of the group of pure ZIF-8 with ultrasonic treatment (Figure 4D). These results may be related to the advantages of the sono-responsive material ZIF-8, which has a high porosity and high specific surface area; this material can increase the efficiency of loading of the sonosensitizer, has excellent adsorption performance for gas, and can enhance the ultrasonic cavitation effect. The enhanced ultrasonic cavitation effect can not only promote the production of ROS but also strengthen the mechanical destruction of the bacterial cell membrane and intracellular components (Hiraoka et al., 2006; Yildirim et al., 2017). In addition, the good antibacterial property of Zn^2+^ released from ZIF-8 can further improve the sustained antibacterial ability of the material (Liang et al., 2021). Therefore, in consideration of the ROS production efficiency and bactericidal effect, ZIF-8 is a good carrier material for HMME, and HMME@ZIF-8 may be a new good sonosensitizer material for sonodynamic antibacterial therapy.

### 3.4 *In vitro* drug release rate studies

In the current study, Met was directly loaded into GelMA hydrogels, the release of Met was close to 70% on the first day, and the cumulative release rate was close to 99% in the first 3 days (Figure 3G). BMP-2 was first loaded into PLGA and subsequently incorporated into the hydrogels to slow the release rate of BMP-2. When considering the cost, the model drug BSA is often used to simulate the release of protein drugs in drug delivery systems. According to previous studies, the model drug BSA was used in this study to replace BMP-2 for drug release experiments (Li D. et al., 2022). BSA was first loaded into PLGA and subsequently mixed into the hydrogels. Approximately 30% of the BSA was released on the first day, after which the release period stabilized for more than 2 weeks (Figure 3G). Met was directly mixed in the hydrogel, which exhibited a typical burst release phenomenon in the first 2 days. The diffusion of the drug inside the hydrogel is considered to be the main driving force (Miri et al., 2018). The model drug BSA needs to first break through the dissolution and swelling of PLGA microspheres to release into the hydrogel and subsequently release from the hydrogel into the outside environment. Therefore, the dual media of PLGA and the hydrogel would allow the entire drug release process of BMP-2 to occur for more than 2 weeks in this study. The different incorporation manners of Met and BMP-2 into the hydrogel may achieve different drug release patterns (immediate and sustained release). In terms of the release rate of the two drugs, the rapid initial release of Met may achieve early intervention and regulation of inflammation, whereas the sustained release of BMP-2 can create certain favorable conditions for subsequent bone tissue repair.

### 3.5 *In vitro* cytocompatibility and *in vivo* biocompatibility assay

**FIGURE 5:**
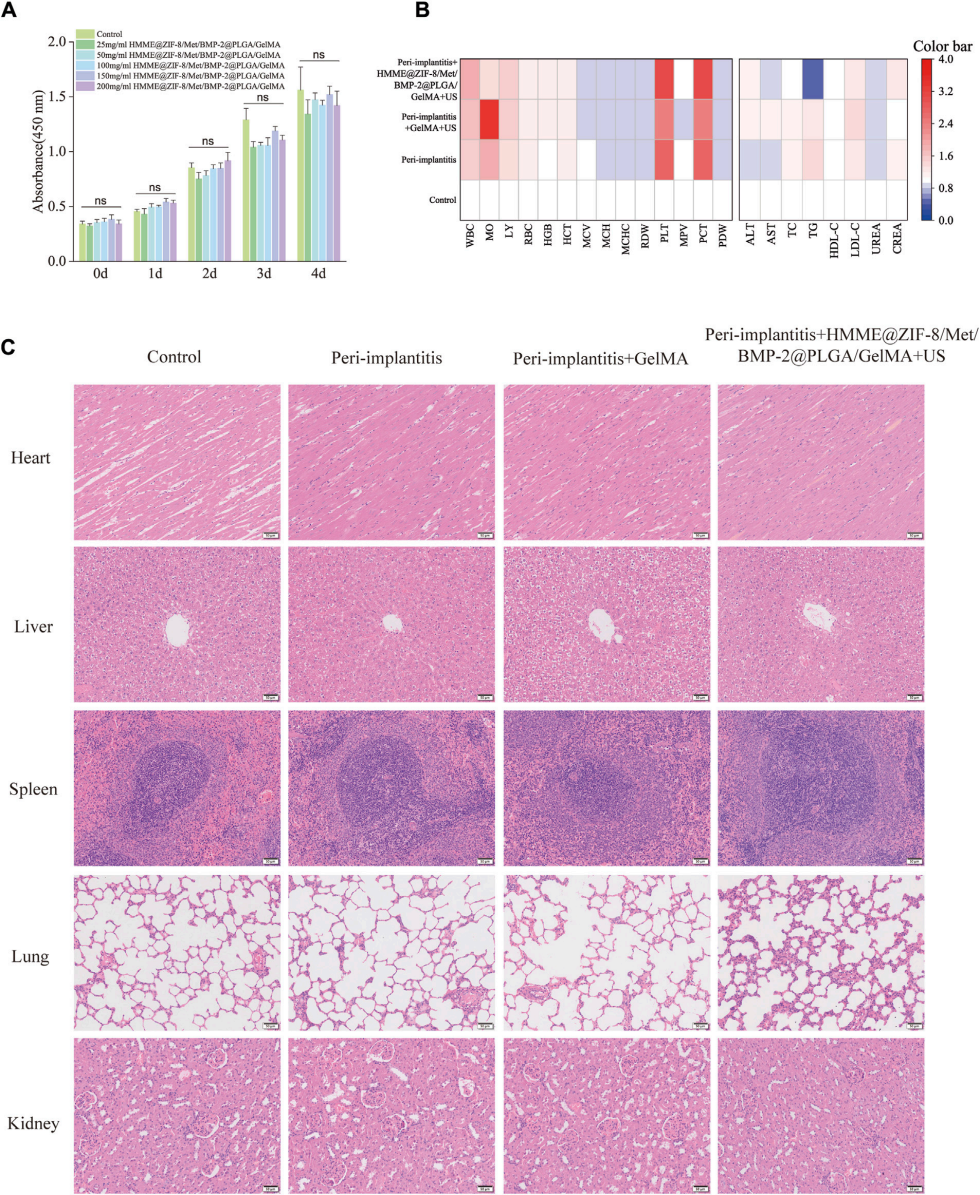
Biocompatibility of the HMME@ZIF-8/Met/BMP-2@PLGA/GelMA composite hydrogels. **(A)** Proliferation of MC3T3-E1 cells cocultured with the extracts of the composite hydrogels for different durations. **(B)** Blood routine analysis and metabolic investigations of rats. **(C)** H&E staining of the major organs of the rats. Scale bar = 50 μm. Data are presented as the mean ± SD (n = 5). Statistical significance is indicated as ns, not statistically significant.

Good biocompatibility is the most fundamental requirement of biomaterials in drug delivery and tissue regenerative engineering. In this study, the CCK-8 assay was used to evaluate the activity of MC3T3-E1 cells cocultured with the extracts of the composite hydrogels at each time point. As shown in Figure 5A, the viability of MC3T3-E1 cells in all of the groups increased normally with time when the composite hydrogels were cocultured with MC3T3-E1 cells for 0, 1, 2, 3, or 4 days, whereas there was no significant difference in cell viability among the groups, thus indicating that the composite hydrogels had good cytocompatibility. For *in vivo* biotoxicity investigation, the blood samples from the tested rats were first tested for any potential systemic toxicity of the composite hydrogels, and the results of routine blood analysis and metabolic investigations were all normal (Figure 5B). Moreover, H&E staining of visceral tissue sections also showed that the composite hydrogels did not cause significant organ damage or pathological changes in major organs (Figure 5C). Collectively, the results demonstrated that the composite hydrogels had good biocompatibility at the cellular and tissue levels.

### 3.6 *In vivo* inflammatory regulation and bone repair analysis

The local structure around implants is complex, and hydrogels have better adaptive properties for filling irregular defects around implants. GelMA hydrogels can cope well with these complex conditions around the implant and function better as biological scaffolds after crosslinking and curing via ultraviolet light. As a good carrier of drugs, it has been widely used in periodontal and implant-related research (Liu et al., 2022). In the current study, HMME@ZIF-8, Met, and BMP-2@PLGA were incorporated into GelMA hydrogels to form HMME@ZIF-8/Met/BMP-2@PLGA/GelMA composite hydrogels, and a bacterial-induced peri-implantitis model in the maxilla of rats was established to detect the effects of the composite hydrogels with adjunctive use of ultrasound on regulating inflammation and promoting bone tissue repair *in vivo*, thus providing a theoretical basis for the application of the sono-responsive nanosystems with ultrasound assistance in peri-implantitis treatment in the future.

**FIGURE 6:**
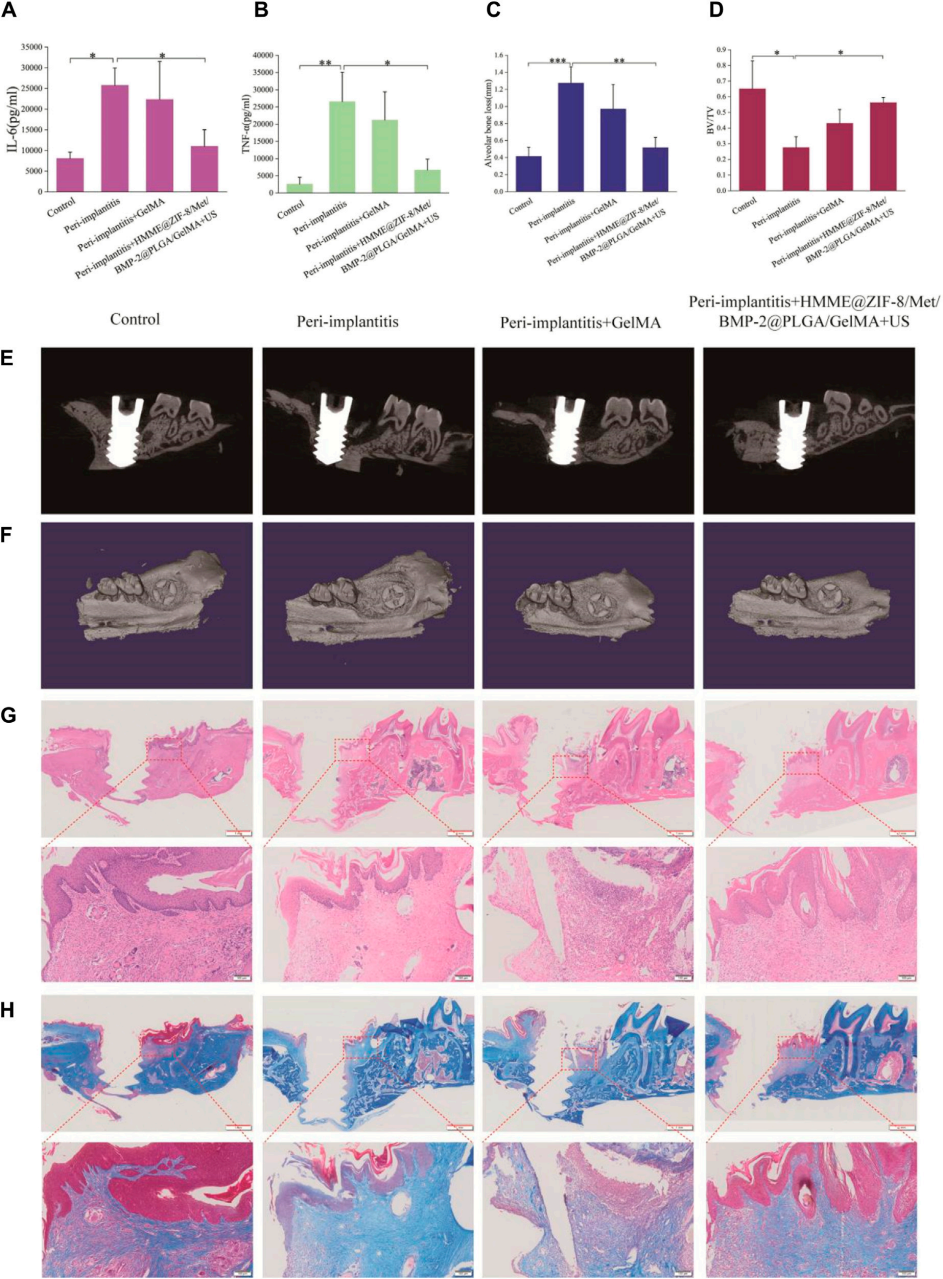
The therapeutic effects of HMME@ZIF-8/Met/BMP-2@PLGA/GelMA composite hydrogels with ultrasound assistance in rats with bacterial-induced peri-implantitis. **(A and B)** Quantification of inflammatory factors (IL-6 and TNF-α). **(C)** Height of alveolar bone loss around the implant. **(D)** Measurement of the bone volume fraction (BV/TV). **(E)** Sagittal micro-CT sectional images of alveolar bone. **(F)** 3D micro-CT images of alveolar bone. **(G and H)** H&E and Masson staining images of decalcified maxillae on the sagittal plane. Scale bars = red: 1 mm, black: 100 μm. Data are presented as the mean ± SD (n = 3). *p < 0.05, **p < 0.01, and ***p < 0.001.

*P. gingivalis* is considered one of the major pathogens of periodontitis and peri-implantitis (Lunar Silva and Cascales, 2021; Alves et al., 2022), and *P. gingivalis-*induced periodontitis and peri-implantitis can lead to bone destruction in periodontal and peri-implant tissues (Reyes, 2021). The injection of pathogenic bacteria around the implant can be used to simulate peri-implantitis in humans, and this model induces inflammation in peri-implant tissues similar to changes observed in human periodontal and peri-implant disease (Koutouzis et al., 2017). Therefore, in this study, a rat model of peri-implantitis was established via local injection of *P. gingivalis* solution around the implant, and the results showed that inflammation and soft and hard tissue destruction were significantly greater in the peri-implantitis group than in the control group (Figure 6).

We first evaluated the expression levels of IL-6 and TNF-α in the gingival tissue of the rats by using ELISA. IL-6 and TNF-α play important roles in the pathogenesis of peri-implantitis (Liu Q. et al., 2023). IL-6 can stimulate osteoclast formation and bone resorption, and the expression of IL-6 is greater in diseased gingival tissues than in healthy tissues (Noh et al., 2013). TNF-α participates in the inflammatory cascade at an early stage to promote the release of other inflammatory mediators, thereby amplifying inflammation (Madianos et al., 2005; Li X. et al., 2019). A significant reduction in the IL-6 and TNF-α levels was detected in the treatment group (Figure 6A, B). This may be related to the following aspects. At the beginning, the HMME@ZIF-8 in composite hydrogels under ultrasound stimulation produces large amounts of ROS, which has a direct killing effect on the peri-implant microflora and blocks the continuous release of virulence factors from pathogenic bacteria. The rapid release of Met from the hydrogels during the early stage may inhibit the inflammatory response of peri-implant tissues. Studies have shown that Met can not only regulate chronic inflammation by improving metabolic parameters but also have direct anti-inflammatory effects (Bharath and Nikolajczyk, 2021); moreover, in different experimental periodontitis rat models, Met has also been found to reduce inflammation, oxidative stress and bone defects (Araujo et al., 2017; Zhao X. et al., 2022; Wang et al., 2023). Our preliminary results suggested promising effects of the composite hydrogels with ultrasound stimulation on regulating inflammation in peri-implantitis.

In our study, micro-CT scanning and histopathological examinations were performed to further analyze the efficacy of the novel therapeutic approach for regulating inflammation and promoting bone tissue repair. Alveolar bone loss around implants is the most typical clinical manifestation of peri-implantitis and can lead to implant loss (Gröbe et al., 2017). Micro-CT is a common method for analyzing alveolar bone loss. Vertical bone loss, which is assessed by measuring the linear distance between the implant neck and the lowest point of the alveolar bone, was used to evaluate alveolar bone destruction around implants (Yin et al., 2023). The results showed that the composite hydrogels with ultrasound stimulation group exhibited significantly less bone destruction than the peri-implantitis group (Figure 6C). The peri-implant bone volume fraction (BV/TV×100%) was also measured, and the results showed that the BV/TV ratio in the treatment group was significantly greater than that in the peri-implantitis group (Figure 6D). Our micro-CT results preliminarily suggested that the new treatment effectively reduced peri-implant bone loss. This effect may be related to the following factors. First, the anti-inflammatory effect of the composite hydrogels with ultrasound stimulation in peri-implantitis may reduce subsequent alveolar bone resorption around the implant. Moreover, BMP-2, which is an important growth factor for osteogenic differentiation and bone regeneration, could be released slowly and was available for a longer duration around the implants in this study. Drug release studies have shown that the drug release process of BMP-2 can last for more than 2 weeks. The sustained release of BMP-2 can create favorable conditions for subsequent bone tissue repair. Subsequently, we conducted H&E and Masson staining to further evaluate the effect of bone repair around the different implants. As shown in Figure 6G, H, inflammatory features, including loosely arranged and unorganized connective tissue and bone destruction, were clearly observed in the peri-implantitis group. In contrast, in the treatment group, the scope and extent of bone and soft tissue destruction were reduced. Collectively, these findings indicate that the composite hydrogels with ultrasound assistance could reduce inflammation and bone loss in peri-implantitis, but the effect on promoting new bone formation requires further extensive studies.

## 4 Conclusion

The results indicated that ZIF-8 was a good carrier material for HMME, and HMME@ZIF-8 demonstrated better ROS production efficiency and antimicrobial efficacy in SDT. Additionally, HMME@ZIF-8 may be a new good sonosensitizer material for sonodynamic antibacterial therapy. HMME@ZIF-8/Met/BMP-2@PLGA/GelMA composite hydrogels combined with ultrasound assistance could reduce the release of the inflammatory factors IL-6 and TNF-α and reduce bone loss around implants in rats with bacterial-induced peri-implantitis. In conclusion, our findings suggest that HMME@ZIF-8/Met/BMP-2@PLGA/GelMA composite hydrogels combined with ultrasound can provide a novel option for treating peri-implantitis in the future.

## Data Availability

The raw data supporting the conclusion of this article will be made available by the authors, without undue reservation.
